# Multi-Institutional Study Evaluating the Role of Early Circulating Tumor DNA Dynamics During Treatment With Immune Checkpoint Inhibitors in Patients With Advanced-Stage Melanoma

**DOI:** 10.1200/PO-25-00254

**Published:** 2026-02-13

**Authors:** Vincent T. Ma, Alice Y. Zhou, Amir Forati, Fauzia Hollnagel, Janmesh D. Patel, Caroline Burkey, Alyssa Steimle, Deepak M. Sahasrabudhe, Matthew C. Mannino, Jennifer L. Schehr, Alexander Birbrair, Shuang G. Zhao, Joshua M. Lang, Adrienne I. Victor

**Affiliations:** ^1^ Department of Medicine, Division of Hematology, Medical Oncology, and Palliative Care, University of Wisconsin-Madison, Madison, WI; ^2^ Department of Dermatology, University of Wisconsin-Madison, Madison, WI; ^3^ Department of Medicine, Division of Oncology, Washington University, St Louis, MO; ^4^ Department of Medicine, University of Wisconsin-Madison, Madison WI; ^5^ Department of Medicine, Division of Hematology/Oncology, University of Rochester, Rochester, NY; ^6^ Department of Human Oncology, University of Wisconsin-Madison, Madison, WI

## Abstract

**PURPOSE:**

The role of circulating tumor DNA (ctDNA) monitoring in predicting treatment response and survival outcomes in early-stage and metastatic solid tumors has shown promising results. The value of early ctDNA dynamics in solid tumors treated with immune checkpoint inhibitors (ICIs) merits further investigation. Our study aims to assess the role of early ctDNA changes, measured 3-4 weeks after initiating ICI therapy, in predicting clinical outcomes in patients with advanced-stage melanoma.

**METHODS:**

We performed a multi-institutional analysis of real-world data from testing patients with unresectable stage III/IV melanoma using a personalized, tumor-informed ctDNA assay (Signatera) during the course of treatment with anti–PD-1-based therapy. ctDNA levels were assessed before the start of treatment and at 3-4 weeks before the second treatment dose. The change in ctDNA (decrease *v* increase) between the two points was assessed to evaluate the odds of objective response and disease control and to evaluate the risk of progression-free survival (PFS) and overall survival (OS).

**RESULTS:**

One hundred seventeen patients were evaluated. Regression analysis revealed that a decrease in ctDNA was associated with increased odds of disease control (odds ratio [OR], 30.56 [95% CI, 10.64 to 87.84], *P* < .001) and objective response (OR, 23.54 [95% CI, 8.58 to 64.57], *P* < .001). Patients with a decrease in ctDNA had an increased PFS (hazard ratio [HR], 0.18 [95% CI, 0.11 to 0.31], *P* < .001) and OS (HR, 0.28 [95% CI, 0.13 to 0.56], *P* < .001) probability.

**CONCLUSION:**

We found that early ctDNA dynamics after only 3-4 weeks of ICI initiation in patients with advanced-stage melanoma appears to be a candidate strategy to predict overall treatment response, risk of progression, and long-term survival. Larger prospective studies are warranted to validate the utility of early ctDNA changes in treatment monitoring.

## INTRODUCTION

Immune checkpoint inhibitors (ICIs) have led to significant improvement in survival outcomes for patients with advanced melanoma over the past decade.^1^ Despite these advancements, a substantial number of patients fail to achieve clinical benefit from ICI therapy.^1,2^ As of 2025, there are four approved classes of ICIs for treatment of advanced-stage melanoma: anti–cytotoxic T lymphocyte-associated antigen 4, anti–PD-1, anti–PD-L1, and anti–lymphocyte-activation gene 3 (anti–LAG-3); with several checkpoint targets undergoing further investigation.^3^ Based on the DREAMseq trial, dual ICI therapy has emerged as the preferred first-line treatment over BRAF-/MEK-targeted therapy with a significant overall survival (OS) benefit in patients with advanced melanoma treated initially with nivolumab/ipilimumab followed by dabrafenib/trametinib compared with the converse sequence (71.8% *v* 51.5% 2-year OS, respectively).^4,5^ Despite therapeutic advancements and better understanding of optimizing treatment sequence, disease progression remains a challenge for many patients with melanoma, underscoring the critical need for robust strategies to assess progression and treatment resistance while on ICI therapy.

**CONTEXT**

**Key Objective**
Does circulating tumor DNA (ctDNA) dynamics at 3-4 weeks inform treatment outcomes in patients with advanced-stage melanoma treated with immune checkpoint inhibitors (ICIs)?
**Knowledge Generated**
ctDNA dynamics at 3-4 weeks after starting ICIs appears to be a biomarker for treatment response and survival outcomes in patients with advanced-stage melanoma. Furthermore, ctDNA clearance during ICI is associated with a favorable treatment outcome.
**Relevance**
Evaluating ctDNA dynamics early in the course of ICI therapy can inform future studies that lead to actionable changes in management to improve treatment responses and survival outcomes for patients with advanced melanoma.

Tumor response to ICI therapy is typically assessed using radiographic imaging at 2-4 months. Early evaluation could guide treatment adjustments to ICI parameters, but predictive methods are limited. RECIST 1.1 remains the standard, mainly in clinical trials, although adapting it to different ICI settings is complex and challenging to apply clinically.^6^ Fluorodeoxyglucose-positron emission tomography/computed tomography (FDG-PET/CT) offers an alternative by assessing both lesion size and metabolic activity. However, imaging has limitations, including false positives, pseudoprogression, high cost, limited access, and slow interpretation, which can hinder timely patient care.^7,8^

Peripheral blood biomarkers offer a potential approach to monitor systemic treatment response in cancer. Historically, lactate dehydrogenase (LDH) has been a negative prognostic marker in melanoma.^9^ In addition, increased levels of interferon gamma, interleukin (IL)-6, IL-10, vascular endothelial growth factor, and hepatocyte growth factor have also been linked to worse outcomes. Higher counts of CD4^+^/CD8^+^ T cells, B cells, and natural killer cells may predict better response to ICIs.^10^ However, many biomarkers, including LDH, can be influenced by nondisease factors such as infection, stress, or treatment toxicity, limiting their reliability.^11^ Currently, no blood-based biomarkers are routinely used in clinical practice to predict or assess response to ICI in advanced melanoma.

Circulating tumor DNA (ctDNA) has recently been recognized as a dependable and more precise biomarker in several solid tumors.^12-14^ The detection of minimal residual disease postsurgery through ctDNA has demonstrated worse disease-free survival and OS in various solid tumor malignancies, including colorectal, pancreatic, breast, ovarian, bladder, and lung cancers.^13,15-20^ Recent studies indicate that ctDNA holds potential as a predictive biomarker for advanced melanoma and other solid tumors, with ctDNA dynamics predicting response to ICI treatment.^22,23^ Furthermore, ctDNA might have the potential to distinguish pseudoprogression from true disease progression in patients with melanoma.^22,24^ However, the role of ctDNA in the context of melanoma treatment monitoring remains exploratory, necessitating further studies to support its predictive capabilities.

Understanding response early in the treatment course for advanced melanoma is crucial for identifying nonresponders to ICIs. This may enable timely implementation of actionable changes in their treatment regimen, aiming to optimize therapeutic strategies and ultimately improve long-term survival outcomes. In this study, we attempt to assess the role of early treatment interval ctDNA changes in predicting disease response and survival outcomes in patients with advanced-stage melanoma treated with ICI therapy.

## METHODS

We retrospectively identified patients with histologically proven unresectable stage III or IV melanoma (per American Joint Committee on Cancer 8th edition) who were treated with anti–PD-1 based therapy (monotherapy or combination ICI therapy) from August 2021 to August 2024 at the University of Wisconsin (Madison, WI), University of Rochester (Rochester, NY), and Washington University (St Louis, MO). All patients participated in treatment monitoring using a commercially available, personalized, exome-based, tumor-informed ctDNA assay (Signatera, Natera, Inc, Austin, TX) with at least two measurements at different time points including one at baseline (before initiation of ICI therapy) and the other at 3-4 weeks after initiation of therapy, but before the second treatment dose.

Patients and data were collected using an electronic medical record system and stored in a RedCap database hosted by the University of Wisconsin. The cohort included patients who were treatment-naïve or previously treated with other systemic agents, including in the adjuvant setting. Patients who were previously treated in the adjuvant setting must have started a new anti–PD-1 based therapy to be eligible for this study.

We used a comprehensive statistical approach to evaluate the associations between early changes in ctDNA levels (decrease *v* increase and percentage change) and various clinical outcomes (treatment response and survival). We evaluated overall treatment response in retrospect by following the largest target lesions using the revised RECIST guideline (version 1.1).^6^ Disease control was defined as stable disease (SD) + partial response (PR) + complete response (CR) with no specified minimum duration of SD. Objective response was defined as PR + CR. Progression-free survival (PFS) was defined as the time from the date of ICI treatment initiation to clinical progression on imaging based on the RECIST criteria or date of death, whichever occurred first. OS was determined based on electronic health record documentation.

Comparisons between patients exhibiting a decrease versus an increase in ctDNA levels at 3-4 weeks post-ICI treatment initiation were made using chi-squared tests for general categorical comparisons. Patients with a net zero ctDNA increase or decrease were categorized alongside the ctDNA decrease.

For contingency tables with expected cell counts <5, Fisher's exact test was used. Continuous variables were summarized as mean ± standard deviation if normally distributed (assessed via the Shapiro-Wilk test and histograms) or median (IQR) if not. Comparisons between ctDNA increase and decrease groups used independent *t*-tests for normal data and Wilcoxon rank-sum tests for non-normal data.

We conducted logistic regression analyses to evaluate the odds of achieving an objective response (CR + PR) and overall disease control (CR + PR + SD), using the change in ctDNA levels (decrease *v* increase) as the primary independent variable. We calculated odds ratios (ORs) along with 95% CIs to assess the strength of these associations.

For survival analysis, we used Cox proportional hazards models to assess the impact of ctDNA changes on PFS and OS. Hazard ratios (HRs) with 95% CIs were reported to quantify the relative risk of progression/death (PFS) or death (OS) associated with changes in ctDNA levels. To visually represent the survival distributions, we plotted Kaplan-Meier survival curves. The log-rank test was used to compare the survival curves and test for statistical significance in survival outcomes between the groups. We set the level for all significance testing at 0.05. We conducted all our analyses using STATA version 18 (StataCorp. 2023. Stata Statistical Software: Release 18. College Station, TX: StataCorp LLC).

## RESULTS

One hundred seventeen patients with advanced-stage melanoma with longitudinal ctDNA monitoring were identified and evaluated in our study. Patient characteristics are listed in Table 1.

**TABLE 1.** Demographic Patient Characteristics Stratified by ctDNA Dynamics at 3-4 Weeks

Characteristic	Total, No. (%)	ctDNA Decrease (≤0 change), No. (%)	ctDNA Increase (>0 change), No. (%)	*P*
No. (%)	117 (100.0)	76 (65.0)	41 (35.0)	
Age, years				.978
<65	43 (36.8)	28 (36.8)	15 (36.6)	
≥65	74 (63.2)	48 (63.2)	26 (63.4)	
Sex				.867
Female	44 (37.6)	29 (38.2)	15 (36.6)	
Male	73 (62.4)	47 (61.8)	26 (63.4)	
Melanoma primary				.755
Cutaneous	88 (75.2)	58 (76.3)	30 (73.2)	
Mucosal	9 (8.6)	7 (9.2)	3 (7.3)	
Unknown primary	7 (6.0)	5 (6.6)	2 (4.9)	
Uveal	12 (10.3)	6 (7.9)	6 (14.6)	
Stage (AJCC 8th edition)				.691
III	15 (12.8)	9 (11.8)	6 (14.6)	
IV (M1a)	17 (14.5)	13 (17.1)	4 (9.8)	
IV (M1b)	20 (17.1)	14 (18.4)	6 (14.6)	
IV (M1c)	38 (32.5)	22 (28.9)	16 (39.0)	
IV (M1d)	27 (23.1)	18 (23.7)	9 (22.0)	
Systemic treatment–naïve				.857
Yes	98 (83.8)	64 (84.2)	34 (82.9)	
No	19 (16.2)	12 (15.8)	7 (17.1)	
Treatment				.620
Ipilimumab/nivolumab	67 (57.3)	46 (60.5)	21 (51.2)	
Nivolumab/relatlimab	23 (19.7)	14 (18.4)	9 (22.0)	
Anti–PD-1 monotherapy	27 (23.1)	16 (21.1)	11 (26.8)	
Objective response				<.001
Yes	47 (57.3)	42 (80.8)	5 (16.7)	
No	35 (42.7)	10 (19.2)	25 (83.3)	
Disease control				<.001
Yes	54 (65.9)	46 (88.5)	8 (26.7)	
No	28 (34.1)	6 (11.5)	22 (73.3)	

Abbreviations: AJCC, American Joint Committee on Cancer; ctDNA, circulating tumor DNA.

The median follow-up was 13.4 months. The median age was 64.5 years. Seventy-three (62.4%) were male. The staging distribution included unresectable stage III (n = 15, 12.8%), M1a (17, 14.5%), M1b (20, 17.1%), M1c (38, 32.5%), and M1d (27, 23.1%). Ninety-eight patients (83.8%) were ICI treatment–naïve. Eighty-eight patients (75.2%) had a cutaneous primary, 12 (10.3%) were uveal, nine (8.6%) were mucosal, and seven (6.0%) had an unknown melanoma primary. A total of 57.3% (n = 67) was treated with ipilimumab with nivolumab, 19.7% (23) was treated with nivolumab with relatlimab, and 23.1% (27) was treated with anti–PD-1 monotherapy (either pembrolizumab or nivolumab; Table 1).

The median baseline ctDNA level was 8.51 MTM/mL (0.00-13,537.88 MTM/mL). Median changes in ctDNA from baseline were –5.51 MTM/mL among patients with ctDNA decrease and +13.31 MTM/mL among patients with ctDNA increase. Sixteen patients (13.7%) had an undetectable ctDNA level at baseline. Patients with undetectable ctDNA levels at both baseline and 3-4 weeks (n = 15, 12.8%) were classified in the ctDNA decrease group. No statistically significant differences in patient characteristics were seen between patients with ctDNA decrease (≤0 change) and ctDNA increase (>0 change; Table 1). One hundred and eight patients had ctDNA monitoring beyond 3-4 weeks after ICI therapy. The median number of ctDNA assessments after baseline collection was 4 (range, 1-18). Among those with ctDNA decrease (n = 76), 52 patients (68.4%) had eventual ctDNA clearance during the course of therapy, whereas among those with ctDNA increase (n = 41), nine patients (22.0%) had eventual ctDNA clearance.

### Treatment Response

Baseline ctDNA level was not significantly associated with disease control (OR, 1.00 [95% CI, 1.00 to 1.00], *P* = .987) or an objective response (OR, 1.00 [95% CI, 1.00 to 1.00], *P* = .721; Table 2). Similarly, ctDNA level at 3-4 weeks was not significantly associated with disease control (OR, 1.00 [95% CI, 1.00 to 1.00], *P* = .086) or objective response (OR, 1.00 [95% CI, 1.00 to 1.00], *P* = .165; Table 2). By contrast, ctDNA clearance at 3-4 weeks was significantly associated with both disease control (*P* < .001) and objective response (*P* = .008).

**TABLE 2.** Odds of Objective Response and Disease Control With ctDNA Dynamics at 3-4 Weeks

ctDNA Outcome	Disease Control (PR + CR + SD)			Objective Response (PR + CR)		
	No.	OR (95% CI)	*P*	No.	OR (95% CI)	*P*
Baseline ctDNA	117	1.00 (1.00 to 1.00)	.987	117	1.00 (1.00 to 1.00)	.721
ctDNA at 3-4 weeks	117	1.00 (1.00 to 1.00)	.086	117	1.00 (1.00 to 1.00)	.165
ctDNA clearance at 3-4 weeks	52	1.00 (1.00 to 1.00)	<.001^a^	52	1.00 (1.00 to 1.00)	.008^a^
ctDNA (increase *v* decrease)						
ctDNA increase (>0 change)	41	Ref	Ref	41	Ref	Ref
ctDNA decrease (≤0 change)	76	30.56 (10.64 to 87.74)	<.001^a^	76	23.54 (8.58 to 64.57)	<.001^a^
Percent change^b^						
≥+20%	17	0.03 (0.01 to 0.10)	<.001^a^	17	0.03 (0.01 to 0.11)	<.001^a^
–19.99% to +19.99%	9	Ref	Ref	9	Ref	Ref
≤–20%	75	22.50 (6.57 to 77.05)	<.001^a^	75	14.67 (5.03 to 42.73)	<.001^a^

Abbreviations: CR, complete response; ctDNA, circulating tumor DNA; OR, odds ratio; PR, partial response; Ref, reference; SD, stable disease.

^a^ Statistically significant at *P* < .05.

^b^ Patients with an undetectable ctDNA level at baseline were excluded in the percentage change odds analysis.

Using ctDNA increase at 3-4 weeks as the reference, patients with a ctDNA decrease at 3-4 weeks had a statistically significant increased odds of disease control (OR, 30.56 [95% CI, 10.64 to 87.74], *P* < .001) and objective response (OR, 23.54 [95% CI, 8.58 to 64.57], *P* < .001).

Of the 16 patients with undetectable baseline ctDNA, 15 remained undetectable and one developed detectable ctDNA at 3-4 weeks. In this cohort, the disease control rate was 81.3% (n = 13) and the objective response rate was 68.8% (n = 11). The patients who developed a detectable ctDNA level at 3-4 weeks had progressive disease as their best response.

We identified five patients who had progressive disease as best response, but with the decrease in ctDNA level at 3-4 weeks. The median decrease in ctDNA was 2.13 (0.09-11.9) MTM/mL. Of these five patients, two subsequently demonstrated target lesion reduction consistent with a radiographic PR, indicative of initial pseudoprogression.

Using a minimal percentage change in ctDNA at 3-4 weeks (–19.99% to +19.99%) as the reference, patients with ctDNA increase ≥+20% had a statistically decreased odds of disease control (OR, 0.03 [95% CI, 0.01 to 0.10], *P* < .001) and objective response (OR, 0.03 [95% CI, 0.01 to 0.11], *P* < .001) and patients with a ctDNA decrease of ≤–20% had a statistically increased odds of disease control (OR, 22.50 [95% CI, 6.57 to 77.05], *P* < .001) and objective response (OR, 14.67 [95% CI, 5.03 to 42.73], *P* < .001; Table 2).

**FIG 1.**
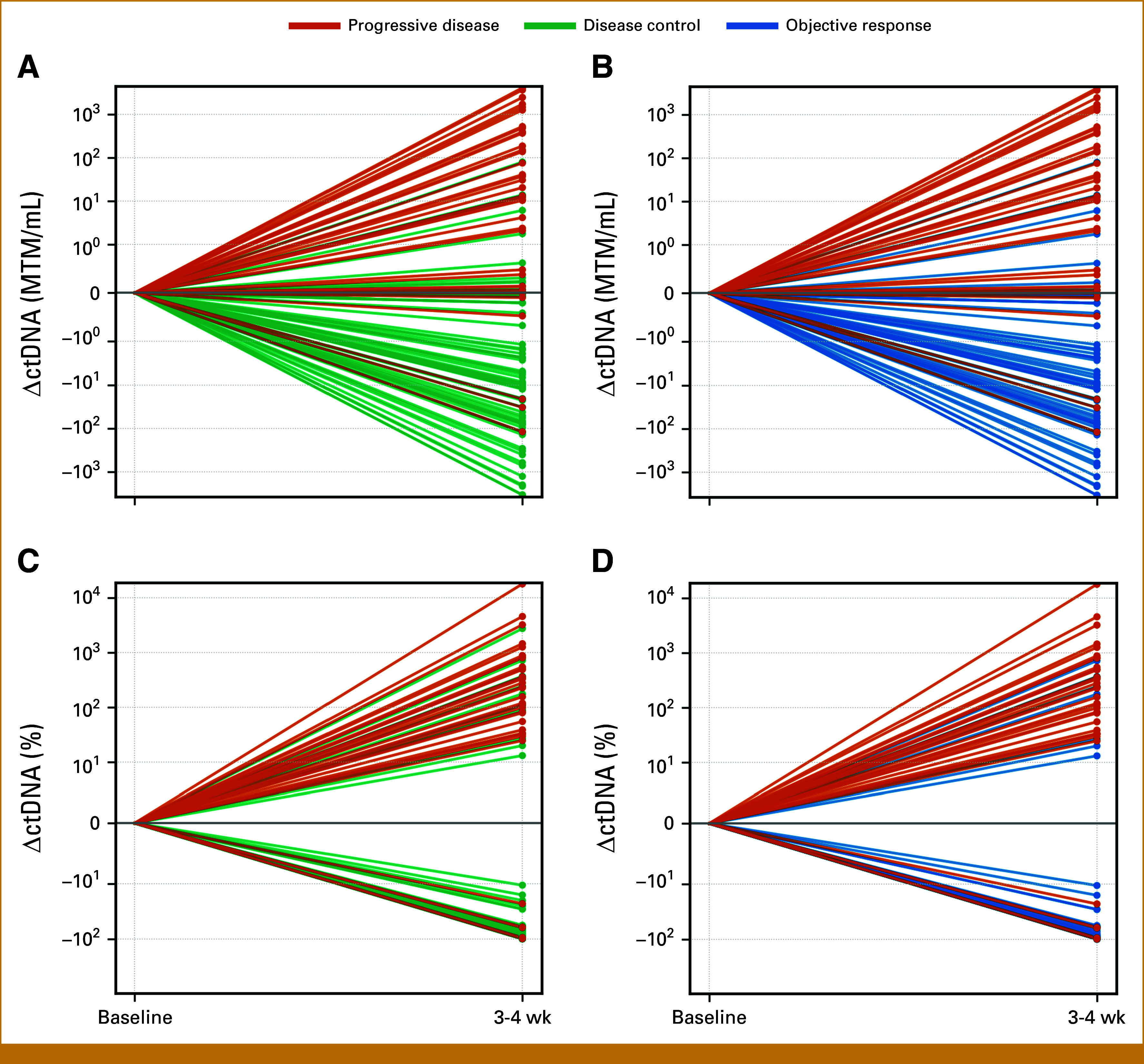
Treatment response based on ctDNA change at 3-4 weeks. (A) ctDNA change (absolute) between disease control and progressive disease; (B) ctDNA change (absolute) between objective response and progressive disease; (C) ctDNA change (percentage) between disease control and progressive disease; (D) ctDNA change (percentage) between objective response and progressive disease. ctDNA, circulating tumor DNA.

Median changes in ctDNA at 3-4 weeks among patients with disease control (n = 79)are –2.34 MTM/mL (–3,401.05 to +79.76 MTM/mL) and –82.11% (–100 to +2,723%) from baseline, whereas the median changes in ctDNA at 3-4 weeks among patients with objective response (n = 70) are –2.53 MTM/mL (–3,401.05 to +79.76 MTM/mL) and –88.32% (–100% to +715%). Median changes in ctDNA at 3-4 weeks among patients with progressive disease (n = 38) are +12.86 MTM/mL (–119.75 to +4,075.84 MTM/mL) and +115.04% (–100% to +17,786.96%) from baseline (Fig 1).

**FIG A1.**
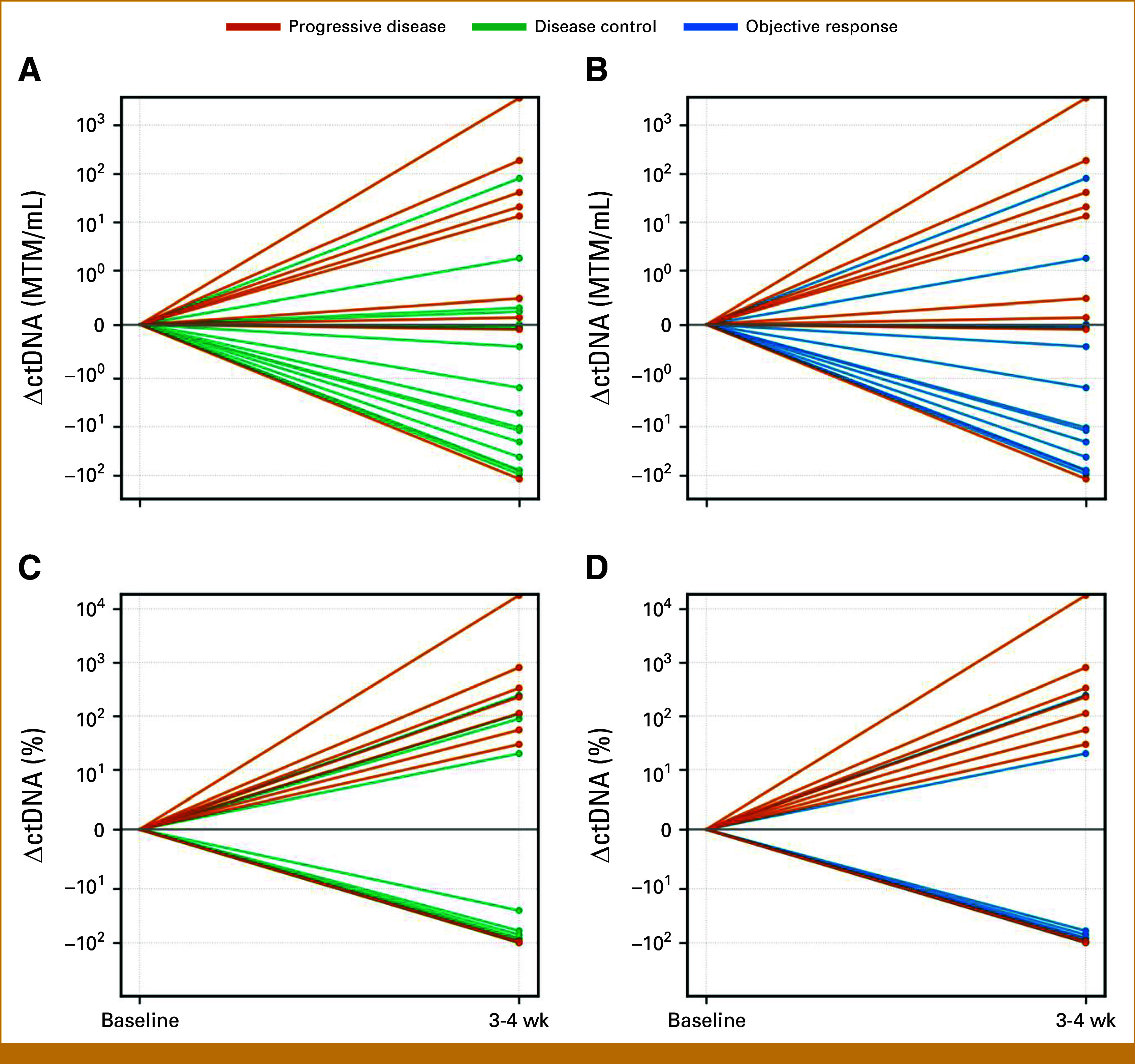
Anti–PD-1 monotherapy (nivolumab or pembrolizumab) treatment response based on ctDNA change at 3-4 weeks (n = 27). (A) ctDNA change (absolute) between disease control and progressive disease; (B) ctDNA change (absolute) between objective response and progressive disease; (C) ctDNA change (percentage) between disease control and progressive disease; (D) ctDNA change (percentage) between objective response and progressive disease. ctDNA, circulating tumor DNA.

**FIG A2.**
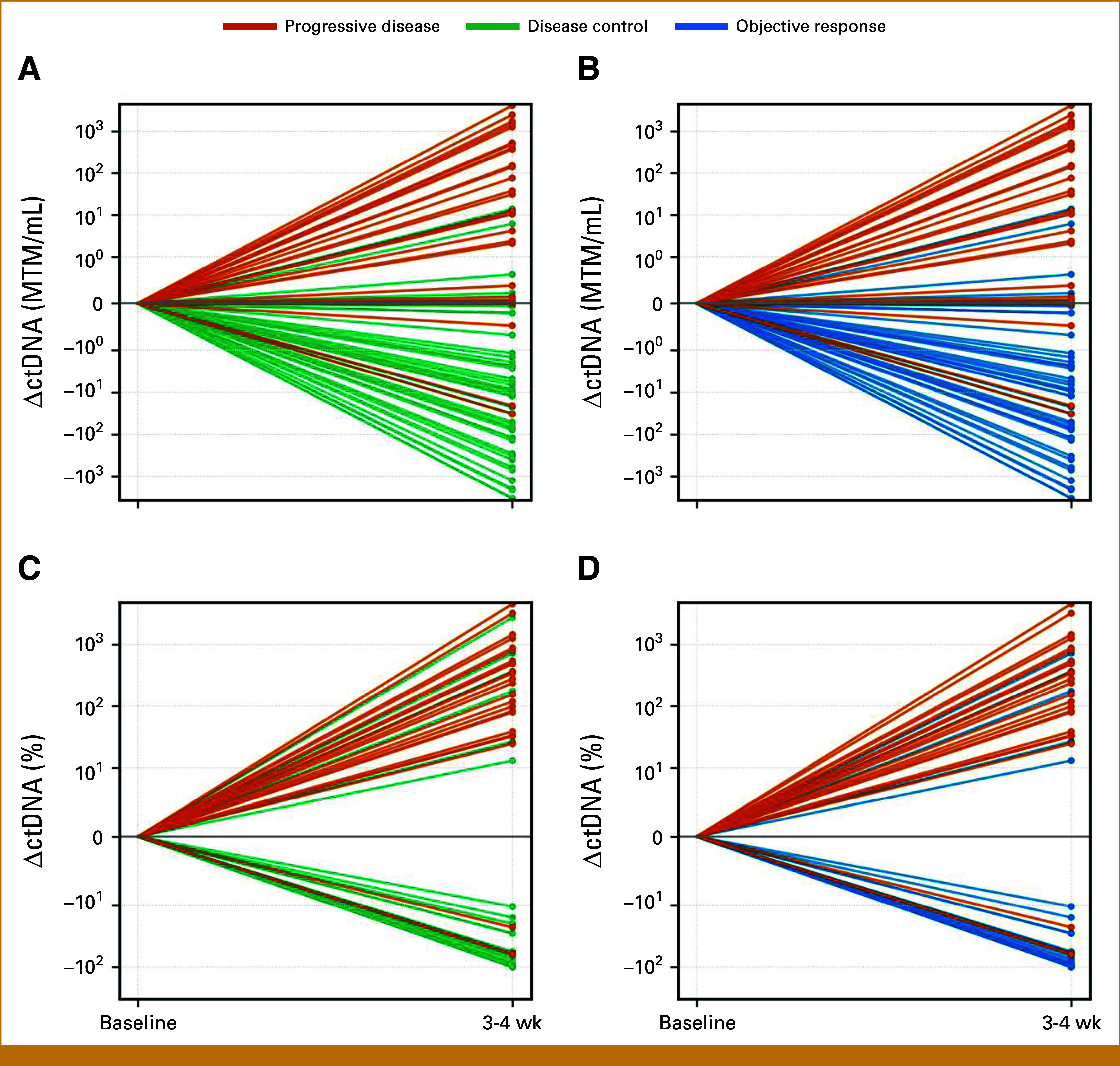
Dual immune checkpoint inhibitor (nivolumab/ipilimumab or nivolumab/relatlimab) treatment response based on ctDNA change at 3-4 weeks (n = 90). (A) ctDNA change (absolute) between disease control and progressive disease; (B) ctDNA change (absolute) between objective response and progressive disease; (C) ctDNA change (percentage) between disease control and progressive disease; (D) ctDNA change (percentage) between objective response and progressive disease. ctDNA, circulating tumor DNA.

Absolute and percentage ctDNA changes at 3-4 weeks were evaluated for patients receiving anti–PD-1 monotherapy (Appendix, Fig A1) and dual ICI therapy (Appendix Fig A2). Interpretation is limited by the small monotherapy cohort (n = 27), but several dual ICI patients showed >2-log ctDNA reductions, which were not seen with anti–PD-1 monotherapy.

### Survival Estimation

Baseline ctDNA levels significantly correlated with PFS (HR, 1.00 [95% CI, 1.00 to 1.00], *P* = .032) and OS (*P* = .005), whereas ctDNA at 3-4 weeks also correlated with PFS (*P* < .001) and OS (*P* < .001; Table 3). Similarly, ctDNA clearance at 3-4 weeks was strongly associated with improved PFS and OS (both *P* < .001).

**TABLE 3.** Risk of PFS and OS With ctDNA Dynamics at 3-4 Weeks

ctDNA Outcome	PFS			OS		
	No.	HR (95% CI)	*P*	No.	HR (95% CI)	*P*-value
Baseline ctDNA	117	1.00 (1.00 to 1.00)	.032^a^	117	1.00 (1.00 to 1.00)	.005^a^
ctDNA at 3-4 weeks	117	1.00 (1.00 to 1.00)	<.001^a^	117	1.00 (1.00 to 1.00)	<.001^a^
ctDNA clearance at 3-4 weeks	52	1.00 (1.00 to 1.00)	<.001^a^	52	1.00 (1.00 to 1.00)	<.001^a^
ctDNA (increase *v* decrease)						
ctDNA increase (>0 change)	41	Ref	Ref	41	Ref	Ref
ctDNA decrease (≤0 change)	76	0.18 (0.11 to 0.31)	<.001^a^	76	0.28 (0.13 to 0.56)	<.001^a^
Percent change^b^						
≥+20%	17	7.25 (2.79 to 18.82)	<.001^a^	17	7.35 (1.71 to 31.47)	.007^a^
–19.99% to +19.9%	9	Ref	Ref	9	Ref	Ref
≤–20%	75	0.76 (0.28 to 2.04)	.596	75	0.41 (0.09 to 1.82)	.241

Abbreviations: ctDNA, circulating tumor DNA; HR, hazard ratio; OS, overall survival; PFS, progression-free survival; Ref, reference.

^a^ Statistically significant at *P* < .05.

^b^ Patients with an undetectable ctDNA level at baseline were excluded in the percentage change hazard analysis.

**FIG 2.**
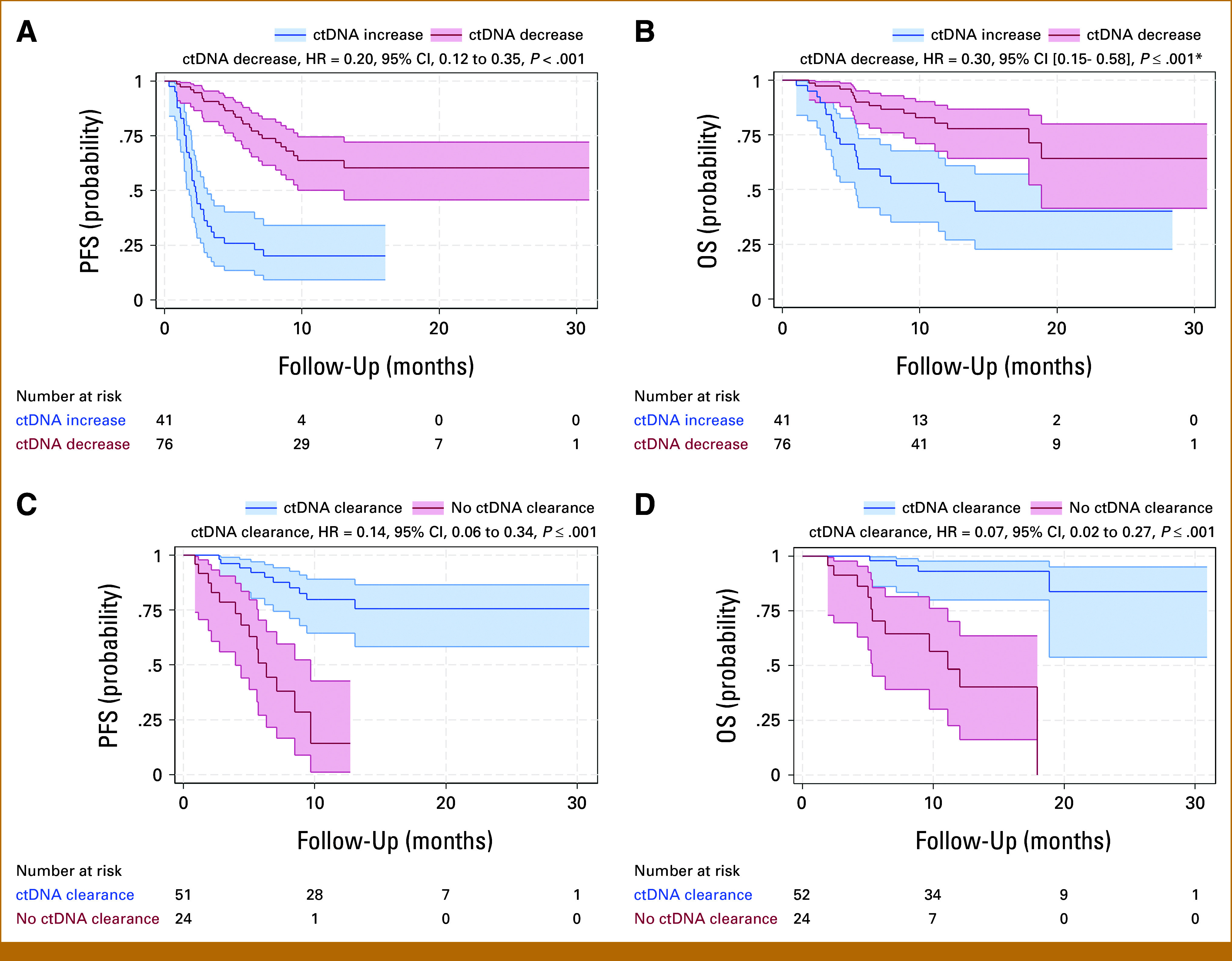
Survival difference between ctDNA increase (>0 change) versus ctDNA decrease (≤0 change) 3-4 weeks after initiation of therapy and ctDNA clearance versus no ctDNA clearance among patients with ctDNA decrease. (A) PFS probability between ctDNA increase (>0 change) versus ctDNA decrease (≤0 change); (B) OS probability between ctDNA increase (>0 change) versus ctDNA decrease (≤0 change). Among patients with ctDNA decrease (n = 76) 3-4 weeks after initiation of therapy, (C) PFS probability between those with eventual ctDNA clearance versus no ctDNA clearance and (D) OS probability between those with eventual ctDNA clearance versus no ctDNA clearance. ctDNA, circulating tumor DNA; HR, hazard ratio; OS, overall survival; PFS, progression-free survival.

Using ctDNA increase at 3-4 weeks as the reference, patients with a ctDNA decrease at 3-4 weeks had a statistically improved risk of PFS (HR, 0.18 [95% CI, 0.11 to 0.31], *P* < .001) and OS (OR, 0.28 [95% CI, 0.13 to 0.56], *P* < .001; Table 3). Among patients with ctDNA increase, the 12-month PFS was 23.1% (95% CI, 11.5 to 35.3) with a median of 2.3 months and the 12-month OS was 43.5% (95% CI, 24.2 to 59.8) with a median of 10.4 months. By contrast, patients with ctDNA decrease had a 12-month PFS of 67.6% (95% CI, 50.2 to 75.1) and a 12-month OS of 77.5% (95% CI, 68.9 to 83.4), with medians not reached (Figs 2A and 2B).

Using minimal percentage change in ctDNA at 3-4 weeks (–19.99% to +19.99%) as the reference, patients with ctDNA increase ≥+20% had a statistically increased risk of progression/death (HR, 7.25 [95% CI, 2.79 to 18.82], *P* < .001) and death (HR, 7.35 [95% CI, 1.71 to 31.47], *P* = .007; Table 3). ctDNA decreases ≤–5 MTM/mL and ≤–20% from baseline were not significantly associated with PFS or OS, likely reflecting the need for longer follow-up to assess survival benefit.

### ctDNA Clearance Outcomes

**FIG 3.**
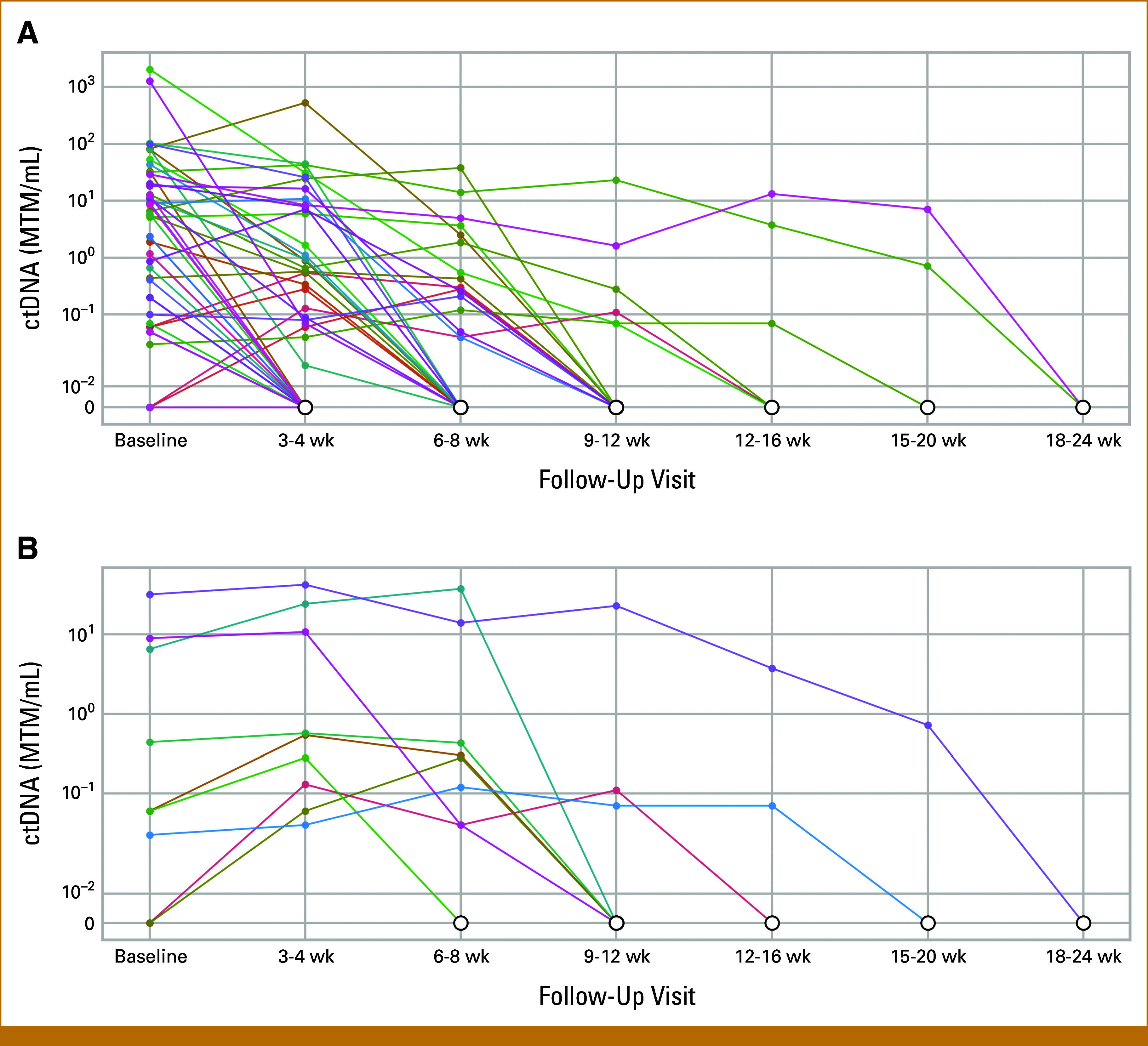
Spider plot of time to first ctDNA clearance during treatment. (A) All patients who eventually had ctDNA clearance (n = 52); (B) all patients who had an initial ctDNA increase before eventual ctDNA clearance (n = 9). ctDNA, circulating tumor DNA.

Among patients with ctDNA decrease (n = 76) 3-4 weeks after initiation of therapy, the survival difference between those with eventual ctDNA clearance (n = 51) versus no ctDNA clearance (n = 25) was evaluated. Patients with eventual ctDNA clearance (undetectable level) had a statistically improved risk of PFS (HR, 0.14 [95% CI, 0.06 to 0.34], *P* < .001) and OS (OR, 0.07 [95% CI, 0.02 to 0.27], *P* < .001; Figs 2C and 2D). The estimated 12-month PFS was 12.8% (95% CI, 2.5 to 38.8) among patient with no ctDNA clearance and 78.6% (95% CI, 69.2 to 88.6) among patients with ctDNA clearance. The estimated 12-month OS was 40.5% (95% CI, 19.8 to 68.3) among patients with no ctDNA clearance and 92.3% (95% CI, 78.2 to 97.2) among patients with ctDNA clearance. Among patients with ctDNA decrease at 3-4 weeks, the majority achieved clearance of ctDNA by weeks 9-12 (Fig 3A).

Of 41 patients with ctDNA increase at 3-4 weeks, nine patients later achieved ctDNA clearance (Fig 3B). Their best responses were CR (n = 2), PR (n = 1), and PD (n = 6) with a median PFS of 1.77 months. Notably, six of these nine patients received adjunct interventions during ICI, including radiotherapy (n = 4) and palliative resection (n = 2) for oligoprogressive or symptomatic disease.

## DISCUSSION

Our study demonstrates that early ctDNA dynamics within 3-4 weeks of ICI initiation predict outcomes in advanced melanoma. A decrease from baseline was associated with a higher odds of disease control and objective response, as well as longer PFS and OS. Among patients with early decreases, subsequent ctDNA clearance further correlated with favorable survival. While previous studies have shown ctDNA's prognostic value, our findings highlight its predictive capability at an earlier interval (3-4 weeks *v* 6-9 weeks).^22,23^

Several liquid biomarkers have been explored for monitoring advanced melanoma, though with mixed reliability. Circulating tumor cells have shown potential but remain limited by challenges in enrichment and isolation.^25-28^ Circulating microRNAs (miRNAs), which are stable in plasma, have been linked to melanoma tumor progression and ICI response^29,30^; for example, increases in miR-1234-3p, miR-4649-3p, and miR-615-3p post-treatment correlated with nonresponse, whereas decreases were associated with CR.^31^ However, low blood concentrations and lack of standardized collection hinder clinical adoption. Thus, despite these promising alternatives, ctDNA remains the most widely available commercial biomarker across tumor types, including melanoma.

Based on our data, the absolute ctDNA level at baseline and at 3-4 weeks does not appear to be predictive for treatment response as compared with PFS and OS. The scientific rationale behind this is not apparent, but raises the possibility that the absolute ctDNA level at a given time point might have a greater impact on the progression-free disease duration or OS interval, rather than achieving the threshold for immediate response outcomes. This is consistent with previous reports showing that objective response rate with ICI therapy does not always correlate with survival as a broader array of factors including molecular, biological, and clinical variables may be other determinants of survival.^33^ While efforts continue to refine ICI response metrics beyond RECIST, including challenges such as pseudoprogression, our results suggest that ctDNA dynamics may be a more reliable indicator of treatment efficacy than absolute levels.

Studies have demonstrated FDG-PET/CT as a novel tool to predict tumor response as early as 1-4 weeks postinitiation of ICI in advanced melanoma.^34,35^ In Hodgkin's lymphoma, PET/CT is already used to guide disease management, enabling treatment continuation or de-escalation based on tumor metabolic activity.^36^ While FDG-PET/CT can measure metabolic activity in tumor lesions, it lacks specificity as uptake can also occur with inflammation, trauma, infection, or immune-mediated toxicities. By contrast, tumor-informed ctDNA provides disease-specific, quantifiable insight into tumor dynamics.^22,23,37,38^

In our study, a minority of patients with transient ctDNA increase had eventual ctDNA clearance from ICI therapy. Although transient disease flare at 3-4 weeks after ICI is possible, the likely rationale for the clearance in our study may reflect the addition of adjunct therapies or interventions. If these interventions could be accounted for in prospective studies, future trials could explore the feasibility of implementing a response-adapted treatment strategy early in the clinical course of advanced melanoma. Based on our study, ctDNA dynamics and clearance appear to be prognostic for ICI outcomes in advanced melanoma, but leveraging ctDNA as a dynamic biomarker to guide therapeutic decisions and optimize patient outcomes could 1 day inform its utility as a predictive biomarker.

While ctDNA holds promise as a tool for guiding cancer therapy and prevention, several challenges persist. Tumor-informed ctDNA assays customize the analysis for individual patients by identifying specific mutations from tumor tissue via next-generation sequencing, thereby enhancing confidence and sensitivity. However, they are hindered by the relatively high turnaround times and cost, and the need for adequate tumor tissue samples.^39^ While ctDNA dynamics show promise as a surrogate biomarker for ICI response, prospective validation is warranted. At present, imaging continues to serve as the standard of care for assessing ICI treatment response. As seen in our study, ctDNA decrease at 3-4 weeks only correlated with radiographic objective response at 80.8% and disease control at 88.5%.

Our study contributes to the growing body of evidence supporting the utility of peripheral blood tumor markers, such as ctDNA, as biomarkers for patients treated with ICIs. With further validation through larger prospective studies and longitudinal follow-up, the integration of early ctDNA changes into treatment decision-making processes may pave the way for response-adapted treatment strategies, ultimately enhancing patient care and outcomes.
